# Lsb1 Is a Negative Regulator of Las17 Dependent Actin Polymerization Involved in Endocytosis

**DOI:** 10.1371/journal.pone.0061147

**Published:** 2013-04-08

**Authors:** Matthias Spiess, Johan-Owen de Craene, Alphée Michelot, Bruno Rinaldi, Aline Huber, David G. Drubin, Barbara Winsor, Sylvie Friant

**Affiliations:** 1 Department of Molecular and Cellular Genetics, UMR7156, Université de Strasbourg and CNRS, Strasbourg, France; 2 Department of Molecular and Cell Biology, University of California, Berkeley, California, United States of America; Institute of Biology Valrose, France

## Abstract

The spatial and temporal regulation of actin polymerization is crucial for various cellular processes. Members of the Wiskott–Aldrich syndrome protein (WASP) family activate the Arp2/3-complex leading to actin polymerization. The yeast *Saccharomyces cerevisiae* contains only one WASP homolog, Las17, that requires additional factors for its regulation. Lsb1 and Lsb2/Pin3 are two yeast homologous proteins bearing an SH3 domain that were identified as Las17-binding proteins. Lsb2/Pin3 that promotes prion induction was suggested to link this prion formation to the actin cytoskeleton. However, the cellular role of Lsb1 and the molecular function of both Lsb1 and Lsb2 remain unknown. In this study, we show that Lsb1 and/or Lsb2 full-length proteins inhibit Las17-mediated actin polymerization *in vitro*, Lsb2 being a less potent inhibitor of Las17 activity compared to Lsb1. Addition of Lsb1 or Lsb2 to the corresponding full-length Lsb1/2 further inhibits Las17 activity. Lsb1 and Lsb2 form homo- and hetero-oligomeric complexes suggesting that these two proteins could regulate Las17 activity via dimerization or cooperative binding. *In vivo*, overexpressed Lsb1 and Lsb2 proteins cluster Las17-CFP in few cytoplasmic punctate structures that are also positive for other Arp2/3-dependent actin polymerization effectors like Sla1 or Abp1. But, only Lsb1 overexpression blocks the internalization step of receptor-mediated endocytosis. This shows a specific function of Lsb1 in endocytosis.

## Introduction

Endocytosis allows the uptake of a wide variety of extracellular molecules and the internalization of the plasma membrane receptors/transporters. The molecular mechanisms of clathrin-mediated endocytosis are conserved from yeast to mammals and the spatio-temporal regulation of the events has been revealed by live-cell imaging [1], [2], [3], [4], [5]. After internalization site selection, an early coat (which includes clathrin and epsin adaptors) assembles that subsequently triggers the invagination of the plasma membrane by recruitment of actin nucleation factors (Sla1 and Las17/WASP) and actin polymerization, its elongation and finally its scission in an endocytic vesicle mediated by the amphiphysins (Rvs161 and Rvs167) [2], [6], [7]. Cargo selection and the early steps of internalization are tightly regulated by ubiquitination [8], [9]. Genetic screens in yeast *Saccharomyces cerevisiae* identified a large number of mutants required for endocytosis (termed *end* mutants) among them several are linked to actin cytoskeleton polymerization (*END3*, *END4/SLA2*, *END5/VRP1*, *END6/RVS161*, *END7/ACT1*, *END9/ARC35*) [10], [11], [12]. The actin cytoskeleton is highly dynamic and therefore requires tight regulation to maintain its integrity. A key cellular factor involved in actin nucleation is the Arp2/3 complex, which needs to be activated by a nucleation-promoting factor (NPF) [13]. Several NPFs, such as the type I myosins Myo3 and Myo5, Abp1, Pan1 and Las17/WASP (Wiskott-Aldrich Syndrome Protein), are required for the internalization step of endocytosis [14], [15], [16], [17].

WASP family members activate the Arp2/3 complex via their carboxy-terminal WCA domain. Additionally they contain an amino-terminal WH1 domain, a GTPase binding domain and a proline rich stretch. The activity of the WCA domain is auto-inhibited by an internal interaction with the GTPase binding domain [18]. This inhibition can be released by the interaction with WASP ligands such as Cdc42, phosphatidylinositol 4,5-bisphosphate (PIP_2_) or several SH3 (Src homology 3) domain-containing proteins [19], [20].

The genome of the budding yeast *S. cerevisiae* encodes for only one WASP homolog, Las17, which is required for normal cell growth, actin cytoskeleton organization and endocytosis [16], [21]. Las17 does not contain the GTPase binding domain and therefore its activity is subjected to another type of specific regulation. On the other hand, numerous SH3 domain-containing proteins have been identified as interacting with Las17 and several have been annotated with a function in endocytosis or actin polymerization regulation (Sla1, Myo3/5, Ysc84, Rvs167, Bbc1, Bzz1) [7], [16], [22], [23], [24]. Most of these interactors act as positive regulators of Las17 activity, however Bbc1 and Sla1 have been identified as negative regulators [25], and their effect is lifted by Bzz1 [26]. Another type of Las17-Arp2/3 complex actin polymerization activity inhibitor is the Syp1 protein, which contains an F-BAR domain and maps to sites of endocytosis [27].

In *S. cerevisiae*, the two highly homologous proteins, Lsb1 and Lsb2 (Pin3) have an SH3 domain, which interacts with Las17 [16]. Lsb1 and Lsb2 are ubiquitinated by the E3 ubiquitin ligase Rsp5, which is involved in many cellular processes such as endocytosis or actin cytoskeleton organization and dynamics [8], [28]. In addition, when overexpressed Lsb2 has been reported to promote [PSI^+^] induction, an ubiquitin regulated process [29], [30]. Lsb2 was shown to colocalize with actin and Cap2 indicating that Lsb2 might link prion formation to the actin cytoskeleton [30]. However the molecular function of Lsb1 and Lsb2 remains unknown and although Lsb1 and Lsb2 are highly homologous, the prion phenotypes were only observed for Lsb2.

Here we show that Lsb1 and Lsb2 are negative regulators of Las17 activity on Arp2/3-complex induced actin polymerization, however only Lsb1 overexpression blocks the internalization step of receptor-mediated endocytosis, suggesting that Lsb1 is involved in endocytosis.

## Results

### Lsb1 and Lsb2 interact *in vivo* with Las17

**Figure 1:**
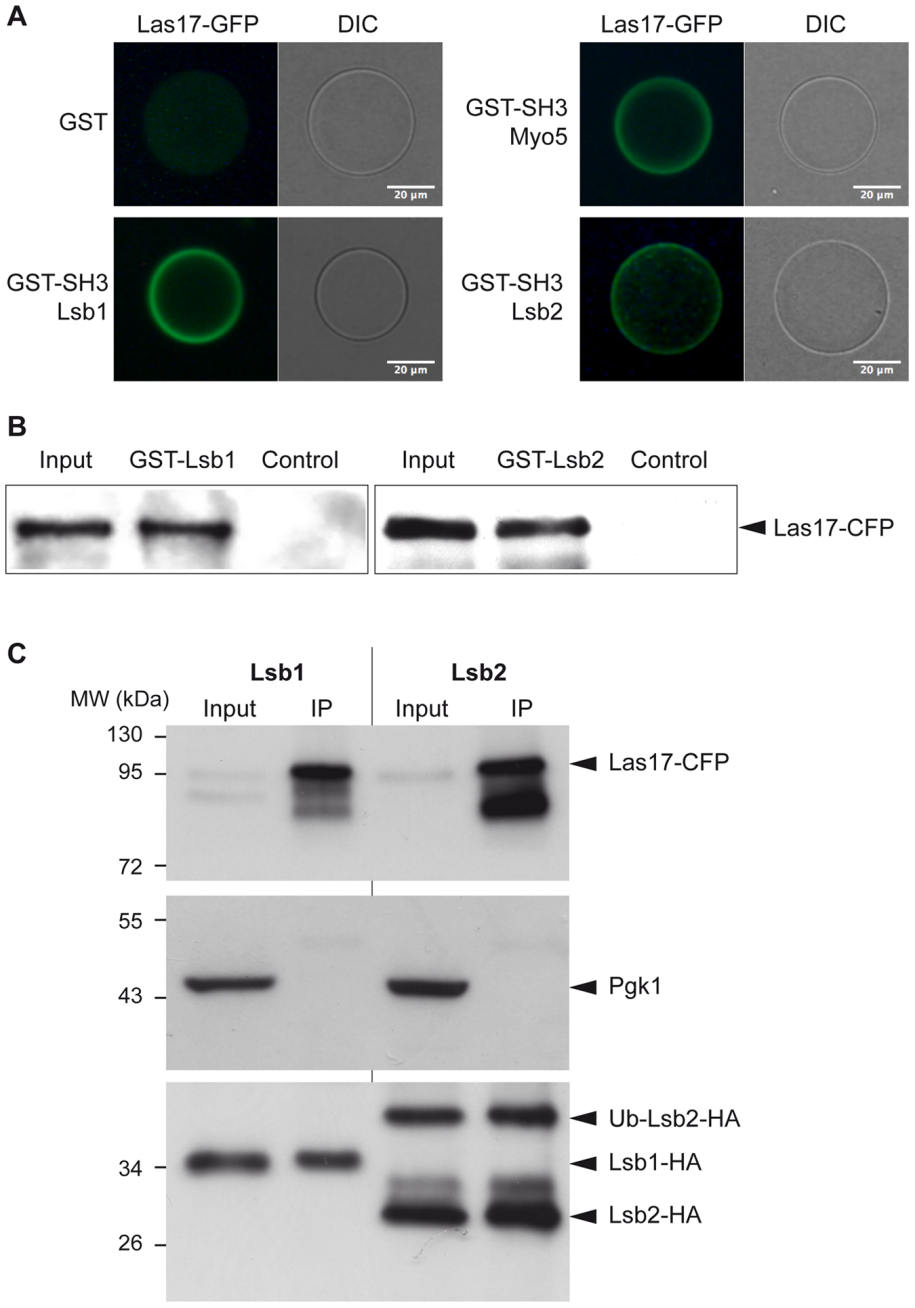
Lsb1 and Lsb2 interact with the WASP Las17. A) GST, GST-SH3-Myo5, GST-SH3-Lsb1 and GST-SH3-Lsb2 coated glutathione-Sepharose beads were incubated with a total protein extract containing Las17-GFP. Beads were analyzed using fluorescence microscopy. GST was used as negative and SH3-Myo5 as a positive control. B) GST-Lsb1 and GST-Lsb2 were expressed in *E. coli* and purified using glutathione Sepharose beads. Beads were incubated with 2 mg of a total protein extract from Las17-CFP expressing cells, washed and analyzed by Western blot. The Input represents the loading of the total protein extract (200 µg). GST was used as negative control. C) A strain expressing Las17-CFP was transformed with plasmids (pUG-HA) encoding for Lsb1-HA or Lsb2-HA. Immunoprecipitation (IP) was performed using rat monoclonal anti-HA antibodies and results analyzed by Western blot using mouse monoclonal anti-GFP, anti-HA and anti-Pgk1 antibodies. The input (40 µg of total protein extract) represents 1/40 of the extract used in the IP experiment (done with 2 mg of total extract).

Previous interaction screens identified the SH3 domain of Lsb1 and Lsb2 as interacting with Las17 [7], [16], [23]. We confirmed this interaction by using the Lsb1 and Lsb2 SH3 domains purified from *E. coli* as GST fusions incubated with a total yeast protein extract containing Las17-GFP. The fluorescent halo around the glutathione-Sepharose beads indicates the interaction between the SH3 domain and Las17-GFP (Figure 1A). Next, we tested the interaction between Las17 and full-length Lsb1 and Lsb2 proteins *in vitro*. We purified the recombinant full-length proteins from *E. coli* as GST fusions and performed a pull down with a total yeast protein extract containing Las17-CFP. The full-length Lsb1 and Lsb2 interacted with Las17 *in vitro* on beads (Figure 1B). This interaction was also observed *in vivo* by co-immunoprecipitation between Las17-HA and Lsb1/Lsb2-Myc tagged proteins [23]. We confirmed this *in vivo* interaction, by using Lsb1-HA and Lsb2-HA tagged proteins expressed in a strain producing Las17-CFP. Las17 was immunoprecipitated by γ-bind Sepharose beads and rabbit anti-GFP serum and the presence of Lsb1 and Lsb2 was assessed by anti-HA immunodetection. The ubiquitously expressed phosphoglycerate kinase Pgk1 was used as a negative control and did not interact with Las17, whereas Lsb1 and Lsb2 proteins coimmunoprecipitated with Las17-CFP (Figure 1C). Lsb2 is ubiquitinated *in vivo* [28], [30] and the ubiquitinated protein also interacts with Las17-CFP. These results confirm that Lsb1 and Lsb2 interact via their SH3 domain with Las17 and are *in vivo* binding partners of Las17.

### Lsb1 and Lsb2 negatively regulate Las17 nucleation promoting activity

Because several proteins, including Myo5, Bzz1 and Las17 have been shown to induce actin polymerization on Sepharose beads *ex vivo* [24], [31], [32], we tested whether Lsb1 and Lsb2 were also able to induce actin polymerization on beads (Figure S1), as they interact with the NPF Las17 (Figure 1). The GST-TH2-SH3^Myo5^ fragment of Myo5 (GST-TH2) was shown to mediate actin polymerization around beads [31]. We incubated GST-TH2-SH3^Myo5^, GST-Lsb1, GST-SH3^Lsb1^, GST-Lsb2 and GST-SH3^Lsb2^ beads with total cell protein extracts and rhodamine-labeled actin (Figure S1). As shown previously [31], the GST-TH2-SH3^Myo5^ fragment mediates actin polymerization as revealed by a fluorescence halo around the beads (Figure S1, GST-TH2). Consistent with the finding by Geli et al. [31], this polymerization was inhibited by addition of the actin depolymerizing agent Latrunculin A (LatA), demonstrating that the halo corresponds to polymerized actin filaments and not to sequestered G-actin. Our results show that this polymerization did not require the presence of Lsb1 or Lsb2 in the cell extract (Figure S1, GST-TH2+*lsb1Δ lsb2Δ* cell extract). In this *in vitro* actin polymerization assay, neither Lsb1, nor Lsb2, or their SH3 domains induce actin polymerization (Figure S1). Moreover, we did not observe G-actin binding for all constructs tested since there was no fluorescent halo around the glutathione-Sepharose beads coated with GST-Lsb1, GST-Lsb2 or their SH3 domains alone (Figure S1). Thus, Lsb1 and Lsb2 interact with Las17 (Figure 1) but do not mediate actin polymerization *in vitro* (Figure S1). However this assay only allows testing for activators of actin polymerization and not inhibitors.

**Figure 2:**
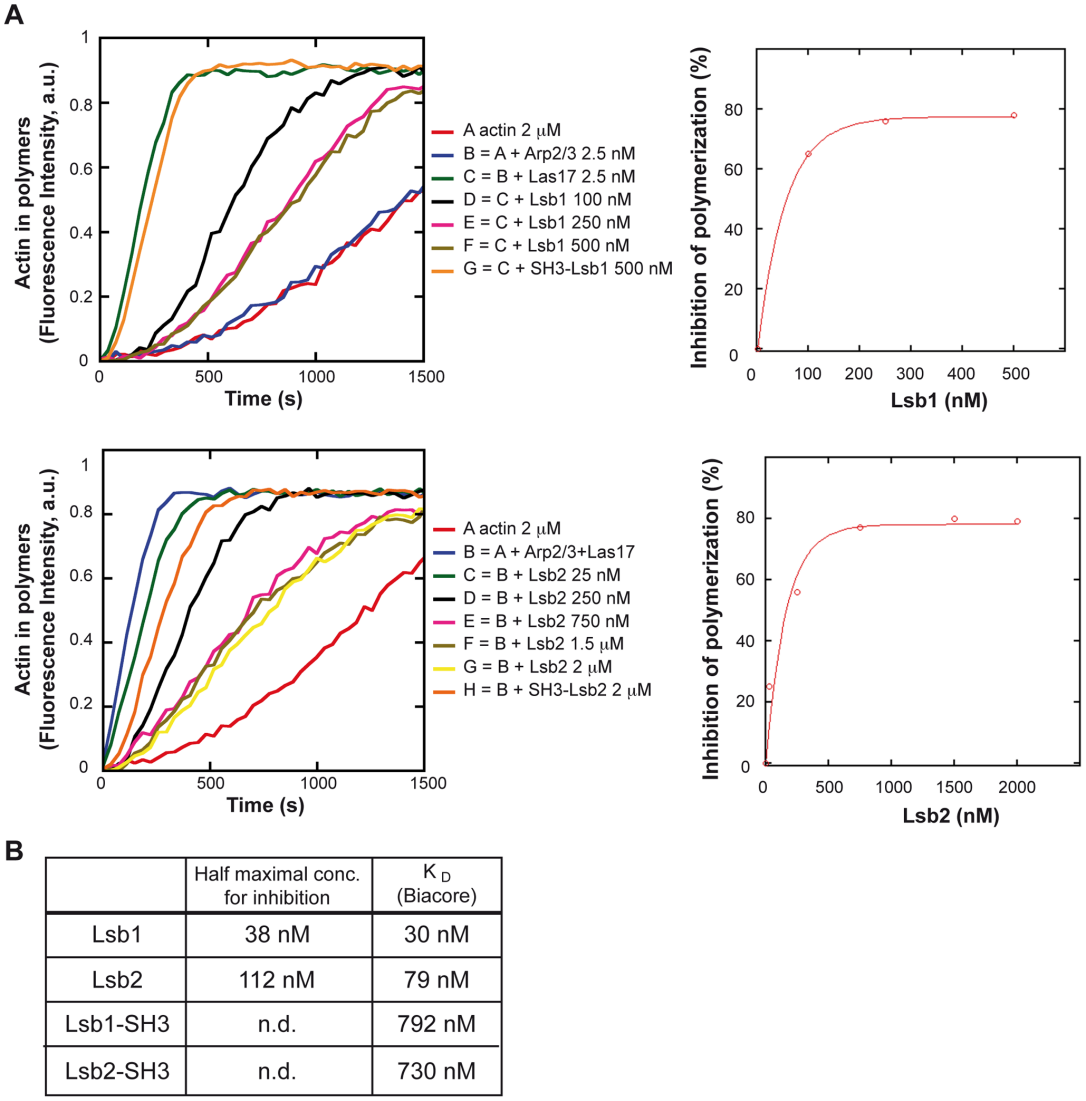
Lsb1 and Lsb2 inhibit Las17 NPF activity. A) A total of 2 µM of rabbit muscle actin (3% pyrene labeled) was polymerized in the presence of the indicated concentrations of purified Arp2/3 complex, Las17, Lsb1, Lsb2, SH3-Lsb1 and/or SH3-Lsb2 recombinant proteins. Actin polymers concentration expressed in arbitrary units (a. u.) was measured by the fluorescence of the pyrene-labeled actin (left panel). Concentration dependence of Las17-Arp2/3-complex induced actin polymerization inhibition by Lsb1 and Lsb2 was calculated from the slope of assembly were the curves are linear (right panel). B) The half maximal concentration for inhibition was calculated by fitting the concentration dependence with a*(1–10∧(-b*x)). The K_D_ values for the interaction between the Las17 protein and the Lsb1, Lsb2, or the SH3 domains of Lsb1 and Lsb2 were determined using a SPR-based assay by Biacore.

Indeed, contrary to the mammalian WASP proteins, Las17 is not auto-inhibited but has to be negatively regulated. Two Las17 inhibitors, Bbc1 and Sla1, have already been described and both contain an SH3 domain [25]. We used a similar pyrene actin polymerization *in vitro* assay as described in Rodal et al. [25] to test the putative inhibitory effect of Lsb1 and Lsb2 and their respective SH3 domains on Las17-Arp2/3-complex induced actin polymerization (Figure 2A and Figure S2). Lsb1, Lsb2 and their SH3 domains were purified by affinity chromatography as GST fusion proteins from *E. coli*, the GST tag was cleaved off and the proteins were further purified by size exclusion chromatography. Purified Lsb1 and Lsb2 proteins had an inhibitory activity on Las17 dependent actin polymerization in a concentration dependent manner (Figure 2A). Saturating concentration of Lsb1 and Lsb2, i.e. 250 nM and 750 nM respectively, inhibited the Las17-Arp2/3-complex induced actin polymerization by 80%, with half maximal concentrations of 38 nM for Lsb1 and 112 nM for Lsb2 (Figure 2). The SH3 domain of Lsb1 and Lsb2 interact with Las17, however on its own the SH3 domain of Lsb1 did not inhibit Las17 NPF activity, whereas the SH3 domain of Lsb2 shows a minimal inhibitory effect on Las17 (Figure 2A and Figure S2). These results were further confirmed by measuring the K_D_ constants of their interaction with Las17 (Figure 2B). Indeed, the Lsb1 and Lsb2 proteins had a much higher affinity for Las17 compared to their SH3 domains since the K_D_ constants measured by a surface plasmon resonance based assay on Biacore3000 were 30 nM and 79 nM for Lsb1 and Lsb2, and 792 nM and 730 nM for their SH3 domain, respectively (Figures 2B). It is noteworthy that the respective K_D_ constants of Lsb1 and Lsb2 are in the range of their K_I_ on Las17-Arp2/3-complex induced actin polymerization.

### Lsb1 and Lsb2 form homo- and hetero-multimers *in vivo*

**Figure 3:**
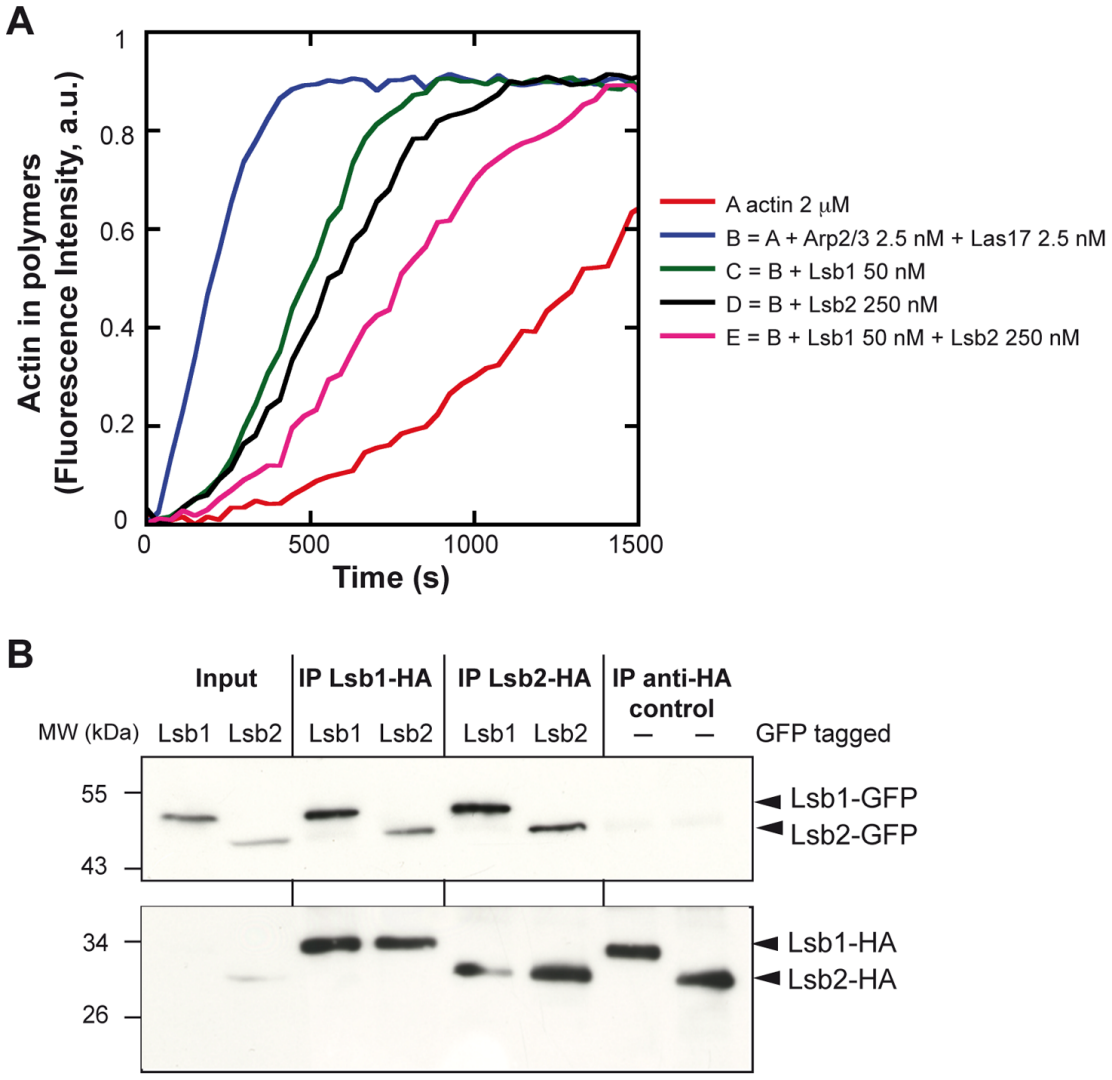
Lsb1 and Lsb2 interact *in vivo*. A) A total of 2 µM of actin (3% pyrene labeled) was polymerized in the presence of indicated concentrations of recombinant purified Arp2/3 complex, Las17, Lsb1 and Lsb2 proteins. B) Wild-type, Lsb1-GFP and Lsb2-GFP strains were transformed with plasmids (pUG-3xHA) expressing Lsb1-HA or Lsb2-HA. Immunoprecipitation (IP) was performed using anti-HA antibodies and results were analyzed by Western blot using anti-HA and anti-GFP antibodies. The wild-type BY4742 strain transformed with the pUG-HA-Lsb1 or -Lsb2 plasmids was used as control. The Input corresponds to the total protein extract after lysis of the Lsb1- or Lsb2-GFP cells.

Since Lsb1 and Lsb2 inhibit Las17-Arp2/3 dependent actin polymerization, we combined both proteins in the actin pyrene polymerization assay and tested their potential additive effect. The addition of either Lsb1 or Lsb2 to the full-length Lsb1/2 further inhibits Las17 activity (Figure 3A), indicating that they are not competing for binding to Las17 and showing that Lsb1 and Lsb2 have an additive inhibitory effect on Las17 dependent actin polymerization. The co-incubation of the Lsb1 SH3 domain with Lsb1 protein did not significantly enhance the inhibition (Figure S2), whereas addition of the SH3 domain of Lsb2 in the presence of the full-length Lsb2 mediated a stronger inhibition compared to only the full-length Lsb2 protein (Figure S2). This shows that the SH3 domain of Lsb2 has an inhibitory effect on Las17, whereas in the same conditions the SH3 domain of Lsb1 did not. This suggests that the Lsb1/2 full-length proteins could regulate Las17 activity via cooperative binding or dimerization between Lsb1 and Lsb2 proteins. Thus we tested whether Lsb1 and Lsb2 could form homo- and/or hetero-multimers. Lsb1-HA was expressed in a strain expressing either Lsb1-GFP or Lsb2-GFP with the tag integrated at the locus and immunoprecipitated using γ-bind Sepharose beads and anti-HA antibodies. The presence of Lsb1-GFP or Lsb2-GFP was tested by Western blot analysis using anti-GFP antibodies. We show that Lsb1-GFP and Lsb2-GFP are co-immunoprecipitated by Lsb1-HA demonstrating that Lsb1 interacts with both itself and Lsb2 (Figure 3B). A similar experiment was performed with Lsb2-HA expressed in a strain bearing Lsb1-GFP or Lsb2-GFP fusions. Lsb1-GFP and Lsb2-GFP were co-immunoprecipitated by Lsb2-HA demonstrating that Lsb2 interacts with both itself and Lsb1 (Figure 3B). This shows that Lsb1 and Lsb2 proteins form homo- and/or hetero-multimers.

### Lsb1 and Lsb2 colocalize with endocytic proteins

**Figure 4:**
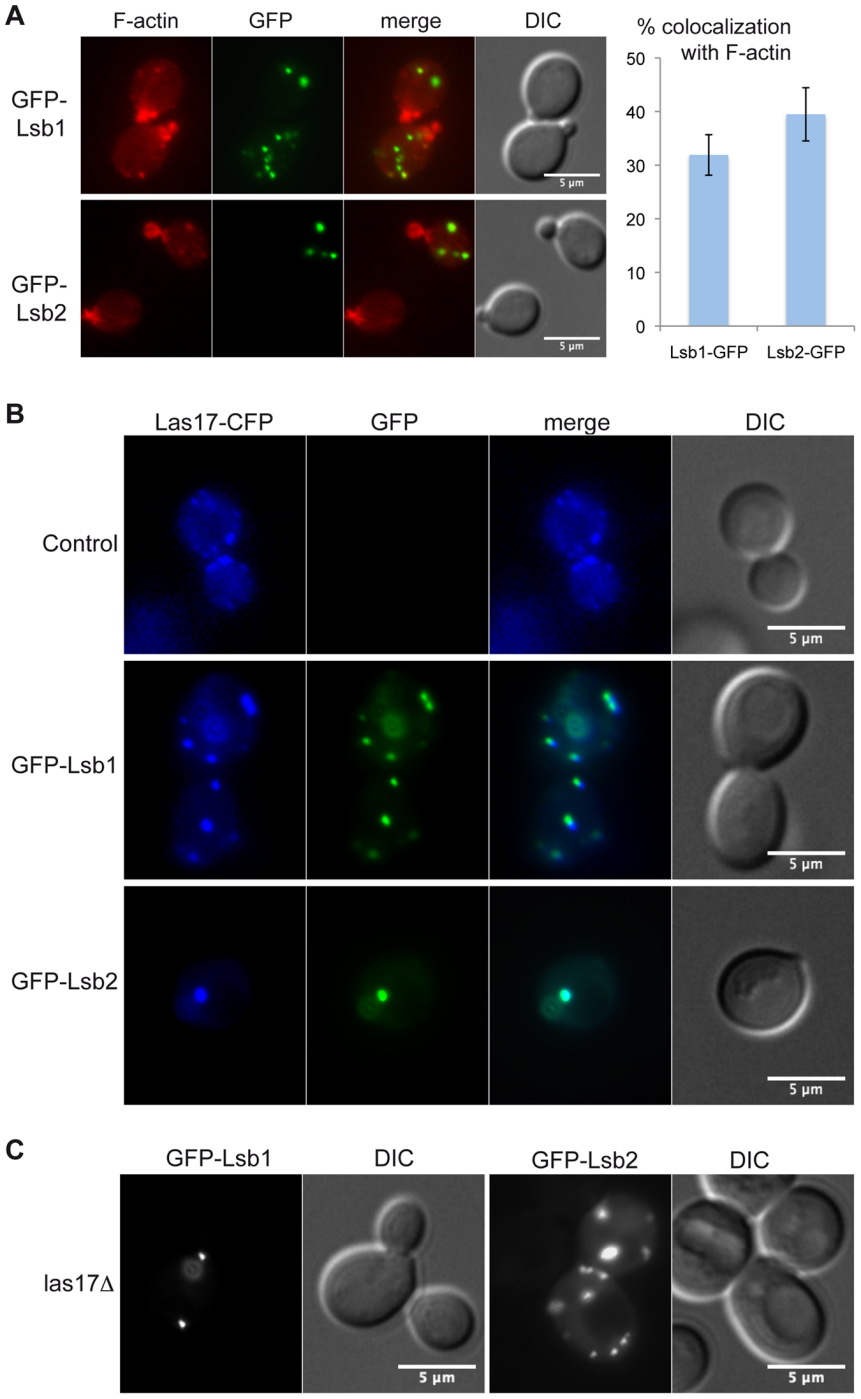
Lsb1 and Lsb2 colocalize with Las17. A) Fluorescence microscopy of GFP-Lsb1 or GFP-Lsb2 expressed from pUG36 vectors in wild type BY4742 cells. The actin cytoskeleton was stained with phalloidin-rhodamine. B) Fluorescence microscopy of GFP-Lsb1 or GFP-Lsb2 (pUG36 vectors) expressed in a Las17-CFP strain. C) Fluorescence microscopy of GFP-Lsb1 or GFP Lsb2 (pUG36 vectors) expressed in a *las17*Δ strain.

Overexpressed Lsb1 and Lsb2 partially colocalize with the actin patch protein Cap2 [30]. Endogenously GFP-tagged Lsb1 and Lsb2 show diffuse cytoplasmic localization [33]. We overexpressed Lsb1 and Lsb2 as N-terminal GFP fusions under the control of the *MET25* promoter and the cells were stained with rhodamine-phalloidin to visualize the actin cytoskeleton (Figure 4A). This F-actin staining confirmed the partial colocalization of GFP-Lsb2 with actin patches (39.5% overlap between GFP-Lsb2 and F-actin) that was previously observed by Chernova and collaborators [30]. We could also observe a partial colocalization between GFP-Lsb1 and F-actin (31.9% overlap between GFP-Lsb1 and F-actin). Lsb1 and Lsb2 interact with and inhibit Las17, so we tested if they colocalized with Las17 in an endogenously CFP-tagged Las17 strain. Overexpressed GFP-Lsb1 and GFP-Lsb2 not only colocalized with Las17-CFP, but clustered Las17-CFP into few intracellular punctate structures (Figure 4B). The punctuate localization of overexpressed GFP-Lsb1 or -Lsb2 does not depend on their interaction with Las17, because intracellular puncta of GFP-Lsb1 and GFP-Lsb2 were also observed in the *las17Δ* mutant cells (Figure 4C). We analyzed whether other actin patch proteins also localized to these clusters and if an interaction with the SH3 domain was required. We tested two SH3-domain containing proteins interacting with Las17, the actin binding protein Abp1 and the endocytic adaptor protein Sla1 [2], [34]. These two proteins act as Arp2/3 dependent actin polymerization regulators, Sla1 inhibits Las17 NPF activity [25], and Abp1 stimulates Arp2/3 dependent actin polymerization and decreases Las17 nucleation-promoting activity [17], [35]. We show that Abp1-GFP interacted with the SH3 domain of Lsb1 and Lsb2 (Figure S3A) whereas the Sla1-GFP protein did not (Figure S4A). Interestingly, both Abp1-CFP and Sla1-mCherry colocalized only partially with GFP-Lsb1 and GFP-Lsb2 (Figure S3B and S4B). Indeed, contrary to Las17-CFP, which was fully clustered by Lsb1- and Lsb2-GFP (Figure 4A), these two proteins still displayed unclustered puncta upon overexpression of Lsb1 or Lsb2. This shows that overexpressed Lsb1 and Lsb2 proteins not only cluster Las17 but also other proteins involved in the Las17-Arp2/3 induced actin polymerization in these aggregates and this independently of their direct interaction with the SH3 domain of Lsb1 and Lsb2.

The molecular function of Lsb1 and Lsb2 as regulators of Las17 activity implies that *in vivo* these proteins should be localized at sites of Las17-dependent actin polymerization. A recent study mentions that Lsb1-GFP and Lsb2-GFP fusion proteins expressed from chromosomal endogenous promoter are detected in the cytoplasm and as single puncta in 10–20% of the cells [30]. We could reproduce this result for Lsb2-GFP that was found as a single puncta in 15% of the cells (n = 324), whereas in our analysis Lsb1-GFP proteins were detected as puncta in 27% of the cells (n = 328) (Figure S6A). However, since Lsb1-GFP and Lsb2-GFP proteins were difficult to visualize due to the weak intensity of their fluorescent signal (Figure S6A), we also tagged them with three copies of GFP (3xGFP) to increase the brightness of the signal (Figure S6B). The yeast cells expressing Lsb1-3xGFP and Lsb2-3xGFP under their own promoter were functional for endocytosis as monitored by proper uptake of the fluorescent dye FM4-64 (Movies S1 and S2). Lsb1-3xGFP was localized as a single puncta near the plasma membrane in 58% of the cells (n = 387), whereas Lsb2-GFP displayed a strong cytoplasmic staining with only 12% of the cells (n = 341) having a single puncta. Moreover, time-lapse imaging of Lsb1-3xGFP revealed that the puncta were highly mobile structures (Movie S1), whereas in the same conditions Lsb2-3xGFP puncta had reduced mobility (Movie S2).

### Clustering of Lsb1 blocks endocytic internalization of Can1

**Figure 5:**
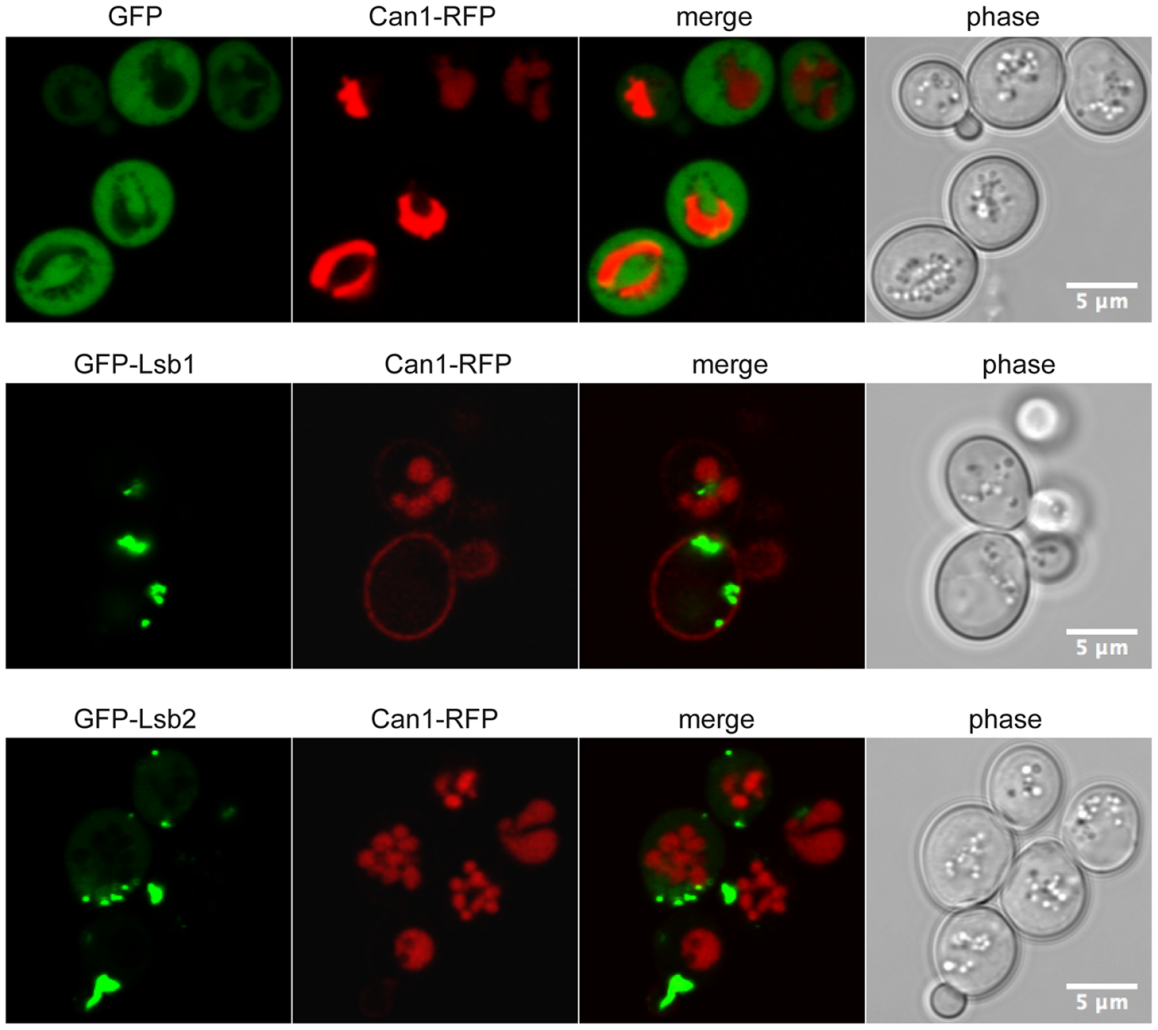
Overexpression of Lsb1 blocks the endocytosis of Can1-RFP. Wild-type BY4742 yeast cells co-transformed by pUG36 (GFP), pUG36-Lsb1 (GFP-Lsb1) or pUG36-Lsb2 (GFP-Lsb2) plasmid and YCplac111-Can1-RFP (Can1-RFP) vector were grown over-night at 30°C in the presence of arginine (5 mM) to induce the endocytic internalization of the arginine permease Can1-RFP, prior their observation by confocal laser scanning microscopy.

To determine the cellular function of Lsb1 and Lsb2 protein apart from the specific role of Lsb2 in prion induction [30], we first analyzed whether the single and double deletion of *lsb1Δ* and *lsb2Δ* genes affects the actin cytoskeleton polymerization (Figure S5A), the intracellular localization of Las17 (Figure S5B) or the uptake of the endocytic dye Lucifer yellow (Figure S5C). None of these actin dependent functions were altered by the deletion of the *LSB1* and/or *LSB2* genes. However these experiments are qualitative and only detect strong defects in endocytosis or actin cytoskeleton organization. The inhibitory function of Lsb1 and Lsb2 on Las17 and their colocalization with actin, Las17, Sla1 and Abp1, all effectors involved in the internalization step of endocytosis, prompted us to test whether Lsb1 and Lsb2 affect endocytosis when overproduced [36]. We analyzed the internalization step of endocytosis upon overexpression of Lsb1 or Lsb2 by following the receptor-mediated endocytosis of the arginine permease Can1 [37] (Figure 5). We analyzed Can1-RFP localization after growth in presence of arginine and observed that its internalization was blocked in cells having large GFP-Lsb1 clusters, whereas it was unaffected in cells displaying a weaker production of GFP-Lsb1 or in cells having a strong production of GFP-Lsb2 (Figure 5). This shows a function for Lsb1 in endocytosis, whereas in similar conditions overexpressed GFP-Lsb2 did not affect Can1-RFP receptor-mediated endocytosis.

## Discussion

The yeast *S. cerevisiae* Lsb1 and Lsb2 are two homologous proteins first identified in a screen for proteins interacting with the NPF Las17 [16]. Later, Lsb2/Pin3 was shown to be involved in prion formation by promoting the conversion of Sup35 into its prion form [PSI^+^] [29]. Moreover, this prion induction function of Lsb2 is regulated by Rsp5-mediated ubiquitination and is dependent on its SH3 domain, which is required for interaction with the actin cytoskeleton [30]. There is little information about the cellular role of Lsb1. The overexpressed Lsb1 protein localizes as punctate structures in the cytoplasm and partially colocalizes with Cap2-RFP [30]. Cap2 is an actin filament capping protein, which binds to barbed ends of actin filaments and predominantly localizes to actin patches [38]. These data suggest a link between Lsb1 and the actin cytoskeleton. Here, we report that the molecular function of Lsb1 and Lsb2 is the negative regulation of Las17 induced actin polymerization. Indeed, Las17 is not auto-inhibited like its mammalian homologs WASP/N-WASP but requires different SH3 domain containing proteins, Bbc1, Sla1 and Bzz1, to inhibit its NPF activity [18], [25], [26]. Our data also show that in our overexpression conditions, only Lsb1 and not Lsb2 is involved in the regulation of the internalization step of endocytosis.

The overexpressed GFP tagged Lsb1 and Lsb2 proteins are localized in cytoplasmic punctate structures ([30], Figure 4, S3 and S4) where they are colocalized with Las17 and other actin patch proteins (Figure 4B, S3 and S4). Even though these two proteins do cluster Las17 when overexpressed, their punctuate localization does not depend on their interaction with Las17 (Figure 4C). The Sla1 and Abp1 proteins show only partial colocalization with Lsb1 and Lsb2 (Figure S3 and S4), showing that not all proteins involved in Las17-Arp2/3 dependent actin polymerization are fully clustered by Lsb1 and Lsb2. Neither Lsb1 nor Lsb2 have been found to form prion structures and they displayed a punctate localization in presence of overexpressed Hsp104, a chaperone known to cure most yeast prions [30], suggesting that these clusters do not result from misfolded Lsb1 or Lsb2 protein aggregation. These large intracellular clusters of overexpressed GFP-Lsb1 and GFP-Lsb2 are likely to be abnormal structures that result from homo- and hetero-multimerization of Lsb1 and Lsb2 due to the high expression level of these GFP fusion proteins. Indeed, when Lsb1 and Lsb2 are expressed as GFP (or 3xGFP) fusion proteins under their endogenous promoter, they are localized in the cytoplasm and to cytoplasmic puncta in some cells. Quantification of cells with a fluorescent puncta shows that approximately 50% of the cells display punctate staining patterns of Lsb1-3xGFP compared to only 15% of the cells for Lsb2-GFP. The spots of Lsb1-3xGFP were highly mobile compared to Lsb2-3xGFP suggesting that they could correspond to individual actin (and/or membrane)-associated structures. Interestingly, the overexpression of Lsb1, but not Lsb2, results in a defect in the internalization step of endocytosis. This shows that this endocytic defect displayed upon Lsb1-GFP overexpression is not solely due to the sequestering of Las17 into intracellular puncta. Thus, the protein composition of the Lsb1 and Lsb2 clusters might be different, and they could contain specific proteins required for endocytosis and for prion induction respectively. However, Lsb1 and Lsb2 could also act as heterodimer to regulate the endocytic function of Las17 and overexpressed Lsb2 could fail to block endocytosis by being less effective in Las17 sequestration compared to Lsb1, indeed Lsb2 is a less potent inhibitor of Las17 activity *in vitro*.

Lsb2, actin and several actin-regulating proteins are localized to potential sites of prion formation [30]. The role of Lsb2 is these structures might be to negatively regulate the Las17-dependent actin polymerization, supporting the view of an active contribution of the actin cytoskeleton in prion formation. Further studies will be needed to completely understand this link and identify other factors implicated in the regulation of the actin polymerization.

We propose a mechanism of inhibition where Lsb1 and/or Lsb2 bind via their SH3 domains to Las17 and inhibit Las17-dependent actin polymerization by preventing the interaction of the WCA domain with the Arp2/3 complex by steric hindrance. This explains why we do not observe inhibition by the SH3 domains alone. The addition of Lsb1 or Lsb2 to the corresponding full-length Lsb1/2 further inhibits Las17 activity and Lsb1 and Lsb2 form homo- and hetero-oligomeric complexes suggesting that these two proteins could regulate Las17 activity via dimerization or cooperative binding. However, despite their similar molecular function, Lsb1 is a more potent Las17 interactor and inhibitor than Lsb2 (Figure 3), this probably correlates with its cellular role in endocytosis, a key cellular process found in all cells and requiring tight regulation. In accordance with this, we could only detect a defect in endocytic internalization in cells highly overexpressing Lsb1 but not Lsb2, suggesting that Lsb1 inhibitory function is tightly regulated. Lsb1-GFP expressed under the control of its own promoter is localized to mobile puncta that could be involved in the control of the Las17 endocytic function. Most of the different Las17 inhibitors identified in yeast are detected very early at the sites of endocytosis. Indeed, Syp1 marks the site of endocytosis, and Sla1 and Bbc1 are recruited early in the process of endocytic internalization [2], [27], [39], [40], whether Lsb1 is also recruited early in the process remains to be established.

In conclusion, the two homologues Lsb1 and Lsb2 share the same molecular function as they are both involved in the negative regulation of the Las17/WASP dependent actin polymerization, however they might have a different cellular function as Lsb2 is linked to prion induction, whereas Lsb1 regulates the internalization step of endocytosis, a process that depends on actin polymerization in yeast.

## Materials and Methods

### Strains, media and genetic manipulations

Standard methods were used for cell growth, DNA manipulations and transformations. *E. coli* strain DH5α was used for plasmid propagation and BL21 (Novagen) for protein production. Bacteria were grown in LB media supplemented with the appropriate antibiotic. Yeast cells were grown at 30°C in rich medium (YPD): 1% yeast extract, 2% peptone, 2% glucose or on synthetic medium (SC): 0,67% yeast nitrogen base without amino acids, 2% glucose and the appropriate dropout mix. Strains used in this study are listed in table S1. Las17-CFP originates from a spore derived from a cross between DDY2738 [39] and BY4741 (Euroscarf). GFP strains were obtained by C-terminal insertion of GFP amplified from pYM28 into the genome [41]. The 3xGFP tagged strains were obtained by cloning *LSB1* and *LSB2* in the PBS-3xGFP-HIS3 integrative plasmid [42] to obtain a fragment encoding for LSB1 or LSB2 fused at its C-terminus in frame to a five Ala linker and triple GFP; wild-type BY4741 cells were transformed with this linearized vector and stable His+ transformants were selected and verified for proper insertion at the *LSB1* or *LSB2* locus by PCR. The *lsb1Δ lsb2Δ* strain was constructed by transformation of the BY4742 *lsb2Δ* strain with a *LSB1* gene disruption cassette amplified from plasmid pFA6-HIS3 [43] and selection on SC-His.

### Plasmid construction

Plasmids used in this study are listed in table S1. *LSB1* and *LSB2* were amplified from genomic DNA and cloned between the *Eco*RI and *Xho*I restriction sites of pGEX4T-1 (GE Healthcare) and the *Eco*RI and *Sal*I restriction sites of pUG36 (kindly provided by J.H. Hegemann and U. Güldener). The sequence coding for SH3 domains of Lsb1 (57–109 aa) and of Lsb2 (59–110 aa) were amplified from genomic DNA and cloned between the *Bam*HI and *Eco*RI sites of pGEX4T-1. The pRS416 LSB1-HA and pRS416 LSB2-HA vectors were constructed by inserting between the *Sal*I and *Eco*RI sites of pRS416-HA a fragment containing 500 bp upstream and the open reading frame of *LSB1* and *LSB2*, respectively amplified from BY4742 genomic DNA. The coding sequence of 3xHA was amplified and cloned between the *Eco*RI and *Eag*I sites of pRS416 [44] to obtain pRS416-HA. The pUG LSB1-HA and pUG LSB2-HA plasmids were constructed by replacing the GFP tag between the *Xba*I and *Xho*I site of pUG36 by the LSB1-HA and LSB2-HA fragments PCR amplified from pRS416-LSB1-HA and pRS416-LSB2-HA respectively.

### Protein expression and purification

BL21 cells transformed with the appropriate plasmid were grown to OD_600 nm_ = 0.4. Protein production was induced with 0.2 mM IPTG for 3 h at 30°C. Cells were harvested, washed and resuspended in PBS. Cells were lysed with a Vibra Cell sonicator (Bioblock Scientific). The extract was clarified at 13000 rpm and incubated with glutathione Sepharose 4B beads (GE Healthcare). For GST tag cleavage, beads were washed and resuspended in 20 mM HEPES (pH 7.5), 1 mM EDTA, 50 mM KCl, 2.5 mM CaCl_2_ and thrombin (3000 U/ml, Promega) and incubated overnight at 4°C. Beads were removed and the protein concentration determined by Bradford assay (Carl Roth), then 10 µg recombinant proteins were injected into a HiPrep Sephacryl S400 HR size exclusion column connected to a ÄKTA FPLC (GE Healthcare) calibrated with the LMW and HMW calibration kits (GE Healthcare) and the recombinant proteins were eluted. Their purity was controlled by SDS-PAGE gels stained with the Protein Staining Solution (Euromedex) and their identity was confirmed by mass-spectrometry (Plateforme Protéomique Strasbourg Esplanade). Las17, the Arp2/3 complex and pyrene actin were produced as reported [25], [45], [46].

### GST pull-down and immunoprecipitation

Purified GST-tagged proteins bound to Glutathione-Sepharose beads (Sigma-Aldrich) were mixed with 4 mg of yeast protein extract (prepared as described below) and incubated for 1 h at 4°C. Beads were washed three times using PBS+500 mM NaCl. Proteins were separated by 10% SDS PAGE and analyzed by Western-blot using standard procedures. Las17-CFP protein was detected with anti-GFP IgG fraction from rabbit anti-GFP serum that is suited for the detection of CFP, a variant of the GFP (Rabbit IgG fraction, Invitrogen).

Las17-CFP cells transformed with Lsb1-HA or Lsb2-HA were grown overnight to an OD_600 nm_ of 0.8 and harvested by centrifugation prior to washing with cold PBS. Cells were resuspended in 1 ml lysis buffer (20 mM Tris pH 7.5, 100 mM NaCl, 5 mM EDTA, 1% Triton-X, 1 mM PMSF, Protease Inhibitor Cocktail (Complete Mini-EDTA free, Roche), broken by vigorous shaking (FastPrep, MP Biomedicals) with glass beads and the extract clarified twice for 5 min at 5000 rpm. Prewashed γ-Bind Sepharose beads (GE Healthcare) were incubated for 1 h at 4°C with anti-GFP IgG fraction from rabbit anti-GFP serum that is suited for the detection of CFP, a variant of the GFP (Rabbit IgG fraction, Invitrogen). 30 µl antibody-coated beads were incubated with 2 mg total yeast extract overnight at 4°C. Beads were washed four times with lysis buffer and once with PBS. Proteins were separated by SDS-PAGE and analyzed by Western-blot using standard procedures. The antibodies used are anti-GST (mouse monoclonal, Sigma-Aldrich), anti-HA (mouse monoclonal, Roche), anti-GFP (mouse monoclonal, Roche) and anti-Pgk1 (mouse monoclonal, Invitrogen).

### Beads directed actin polymerization assay

The beads-directed actin polymerization assay was performed as described in Soulard *et. al.* [24]. Briefly, the actin polymerization reaction was initiated by adding 2 to 3 µg of GST fusion protein to 7 µl yeast extract (20 mg/ml), together with 1 µl of ATP-regenerating mix and 1 µl of 10 µM rhodamine-labeled actin from human platelet (Cytoskeleton). After 15 min incubation at room temperature, samples were observed with a fluorescence microscope (Axiovert200, TRITC and DIC filters, Zeiss). As a control Latrunculin A (Sigma-Aldrich) was added to a final concentration of 10 µM prior to addition of the beads.

### Actin polymerization by fluorescence spectroscopy

Actin nucleation was performed essentially as described by Higgs *et. al.* [19]. Briefly, 10 µM monomeric actin was mixed with the relevant proteins at the indicated concentrations and changes in pyrene fluorescence were followed using a MOS450 Bio-Logic fluorimeter (Bio-Logic-Science Instruments). Polymerization was done at room temperature. The rate of polymerization was calculated from the slope of assembly curves at 50% polymerization, where the curves are linear.

### Surface plasmon resonance

Las17 was coupled via an amino group to a CM5 chip in a Biacore3000 (GE Healthcare). The K_D_ constants were determined at 25°C in a HBS-EP buffer using ligand (Lsb1, Lsb2, the SH3 domain of Lsb1 or Lsb2) concentrations between 25 nM and 10 µM (Figure S7).

### Endocytosis assay and microscopy

Yeast cells were grown in the appropriate media over night to OD_600 nm_ = 0.3. The Lucifer yellow staining was done as previously described [12]. The endocytosis assay was done by incubating yeast cells bearing the Can1-RFP plasmid (pFL91 = YCplac111-Can1promoter-CAN1-mRFP, a kind gift from M. Opekarova, [37]) and pUG36-Lsb1 or pUG36-Lsb2 plasmid in SD-leu-ura medium in presence of arginine at 5 mM over night at 30°C, prior their observation at OD_600 nm_ = 0.4 by confocal microscopy (Zeiss LSM700 microscope, Plateforme Microscopie et Imagerie, IBMP, Strasbourg). Actin was stained with TRITC-phalloidin (Invitrogen) as previously described [24]. The Lsb1-3xGFP and Lsb2-3xGFP cells were stained by incubation at 30°C for 10 min with the lipophilic marker of endocytosis FM4-64 (16 µM), the cells were washed two times and observed in SC-his medium 5 min (Lsb1-3xGFP) or 10 min (Lsb2-3xGFP) after the endocytic uptake of the dye. The images were recorded by dual TRITC (FM4-64) and GFP time-lapse fluorescence microscopy and captured at 20-sec time intervals. The display rate is 2 frames per second. For these experiments, yeast cells were observed in the appropriate medium using an epifluorescence microscope (Axiovert200, Zeiss, 100× objective, DIC, TRITC and GFP filters) and images were acquired with the Axiovision (Zeiss) software using the CoolSnapHQ2 camera (Roper Scientific). Images were processed with the ImageJ software (Rasband, W.S., ImageJ, U. S. National Institutes of Health, Bethesda, Maryland, USA, http://imagej.nih.gov/ij/, 1997–2011). The degree of colocalization between GFP-Lsb1 or GFP-Lsb2 proteins and F-actin stained by rhodamine-phalloidin was quantified by calculating the Mander's overlap coefficient [47], using manually adjusted threshold values to remove the cytoplasmic GFP staining of GFP-Lsb1 and GFP-Lsb2, by using the JACoP v2.0 plugin for ImageJ [48].

## Supporting Information

Figure S1 **Lsb1 and Lsb2 do not induce actin polymerization on beads.** A) and B) Glutathione Sepharose beads coated with either GST, GST-TH2,SH3-Myo5, GST-SH3-Lsb1, GST-SH3-Lsb2, GST-Lsb1 or GST-Lsb2 were incubated with total yeast protein extract in the presence of ATP, ATP-regenerating system and rhodamine-labeled actin. Actin polymerization or binding was observed by fluorescent microscopy as a fluorescent halo around the beads. To discriminate between binding and polymerization, 10 µM Latrunculin-A was added before the incubation.
(TIF)

Figure S2 **The SH3 domains of Lsb1 or Lsb2 proteins do not inhibit Las17 activity.** A total of 2 µM of actin (3% pyrene labeled) was polymerized in the presence of indicated concentrations of recombinant purified Arp2/3 complex, Las17, Lsb1, Lsb2, SH3-Lsb1 and/or SH3-Lsb2 proteins. Actin polymers concentration expressed in arbitrary units (a.u.) was measured by the fluorescence of the pyrene-labeled actin.
(TIF)

Figure S3 **Lsb1 and Lsb2 colocalize with Abp1.** A) GST, GST-SH3-Myo5, GST-SH3-Lsb1 and GST-SH3-Lsb2 proteins coated on glutathione Sepharose beads were incubated with a total protein extract from *ABP1-GFP* yeast cells. Beads were analyzed by fluorescence microscopy. GST was used as a negative control. B) The *ABP1-CFP* cells were transformed by pUG36-Lsb1 (GFP-Lsb1) or pUG36-Lsb2 (GFP-Lsb2) plasmids and the cells were observed by fluorescence microscopy.
(TIF)

Figure S4 **Lsb1 and Lsb2 colocalize with Sla1.** A) GST, GST-SH3-Myo5, GST-SH3-Lsb1 and GST-SH3-Lsb2 proteins coated on glutathione Sepharose beads were incubated with a total protein extract from *SLA1-GFP* cells. Beads were analyzed using fluorescence microscopy. GST was used as a negative control. B) The *SLA1-mCherry* strain was transformed by pUG36-Lsb1 (GFP-Lsb1) or pUG36-Lsb2 (GFP-Lsb2) plasmids and the cells were observed by fluorescence microscopy.
(TIF)

Figure S5 **Las17 localized normal in a** ***lsb1*** **Δ** ***lsb2*** **Δ strain.** A) The actin cytoskeleton was stained with phalloidin-rhodamine in wild type, *lsb1*Δ, *lsb2*Δ and *lsb1*Δ *lsb2*Δ cells prior observation by fluorescence microscopy. B) The exponentially growing *LAS17-CFP*, *lsb1*Δ *LAS17-CFP*, *lsb2*Δ *LAS17-CFP* and *lsb1*Δ *lsb2*Δ *LAS17-CFP* cells were observed by fluorescence microscopy. C) Wild type BY4742 and *lsb1*Δ *lsb2*Δ cells were analyzed by fluorescence microscopy after incubation for 60 min with the fluorescent dye Lucifer yellow (LY). LY is internalized via endocytosis and transported to the lumen of the vacuole.
(TIF)

Figure S6 **Lsb1 and Lsb2 expressed under their endogenous promoter localize to punctate structures.** A) The BY4741 Lsb1-GFP and Lsb2-GFP cells were observed by fluorescence microscopy. B) Wild-type cells carrying a chromosomically integrated LSB1-3xGFP or LSB2-3xGFP fusion were analyzed for GFP fluorescence.
(TIF)

Figure S7 **Biacore data of Lsb1 and Lsb2 with Las17.** The raw data of measured K_D_ constants obtained by SPR-based Biacore3000 measurements with Las17 and at different concentrations of (A) Lsb1, (B) Lsb2, (C) SH3-Lsb1 and (D) SH3-Lsb2 purified recombinant proteins.
(PDF)

Movie S1 **The Lsb1-3xGFP cells were stained with the lipophilic marker for endocytosis FM4-64 and observed for GFP (green) and FM4-64 (red) fluorescence 5 minutes after FM4-64 internalization in SC-his medium.** The images were recorded by dual TRITC (FM4-64) and GFP time-lapse fluorescence microscopy and acquired at 20-second time intervals. The display rate is 2 frames per second.
(AVI)

Movie S2 **The Lsb2-3xGFP cells stained with the endocytic lipophilic dye FM4-64 were observed in SC-his medium by fluorescence microscopy 10 minutes after FM4-64 internalization.** The images were recorded by dual TRITC (FM4-64) and GFP time-lapse fluorescence microscopy and acquired at 20-second time intervals. The display rate is 2 frames per second.
(AVI)

Table S1 Strains and plasmids used in this study.
(DOC)
