# Supplementary figures and images for: Lsb1 Is a Negative Regulator of Las17 Dependent Actin Polymerization Involved in Endocytosis
Source: PLoS One. 2013 Apr 8;8(4):e61147. doi: 10.1371/journal.pone.0061147 (PMC3620054; doi:10.1371/journal.pone.0061147)

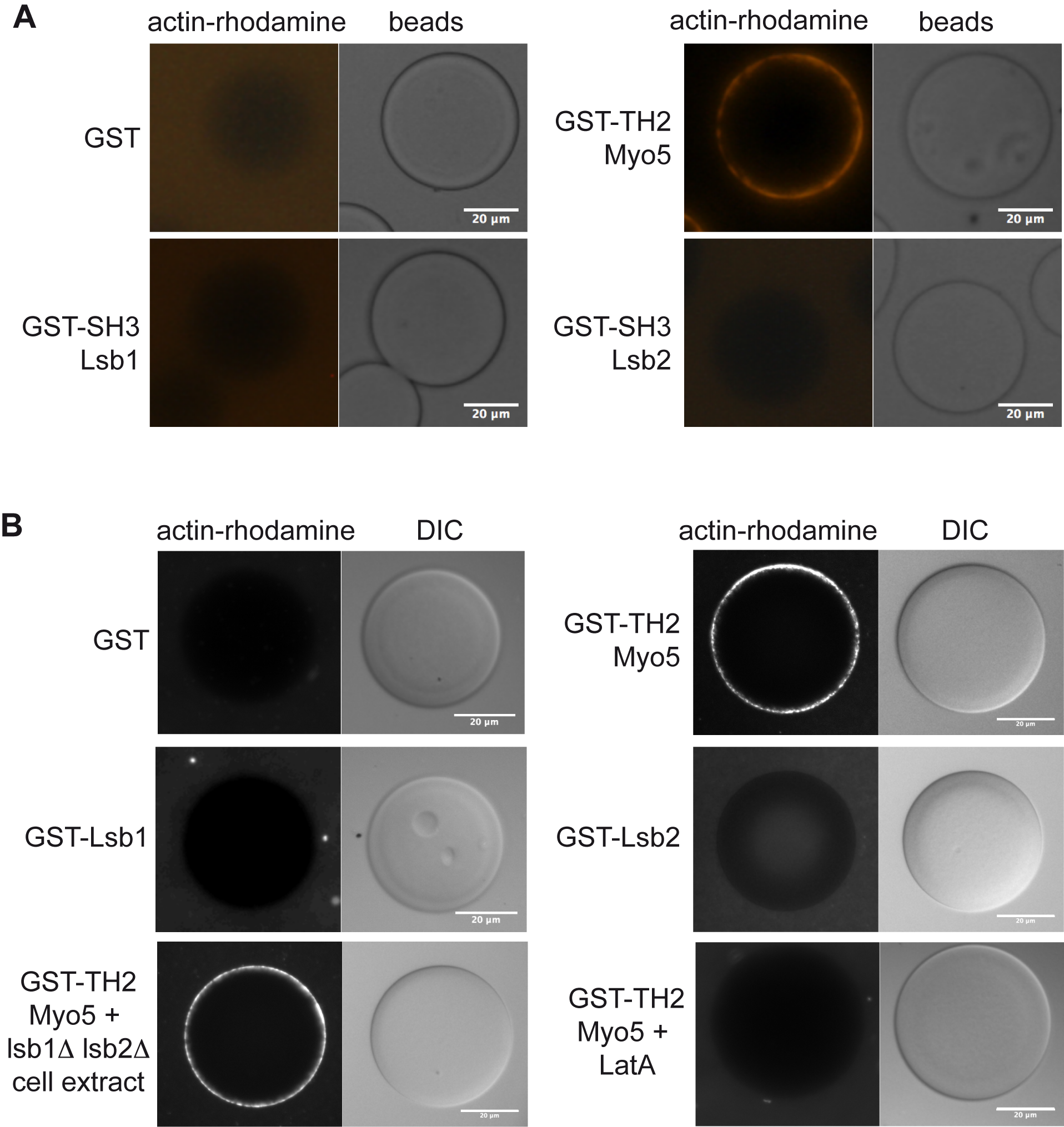

Supplement: Figure S1 — Lsb1 and Lsb2 do not induce actin polymerization on beads. A) and B) Glutathione Sepharose beads coated with either GST, GST-TH2,SH3-Myo5, GST-SH3-Lsb1, GST-SH3-Lsb2, GST-Lsb1 or GST-Lsb2 were incubated with total yeast protein extract in the presence of ATP, ATP-regenerating system and rhodamine-labeled actin. Actin polymerization or binding was observed by fluorescent microscopy as a fluorescent halo around the beads. To discriminate between binding and polymerization, 10 µM Latrunculin-A was added before the incubation. (TIF) [file pone.0061147.s001.tif]

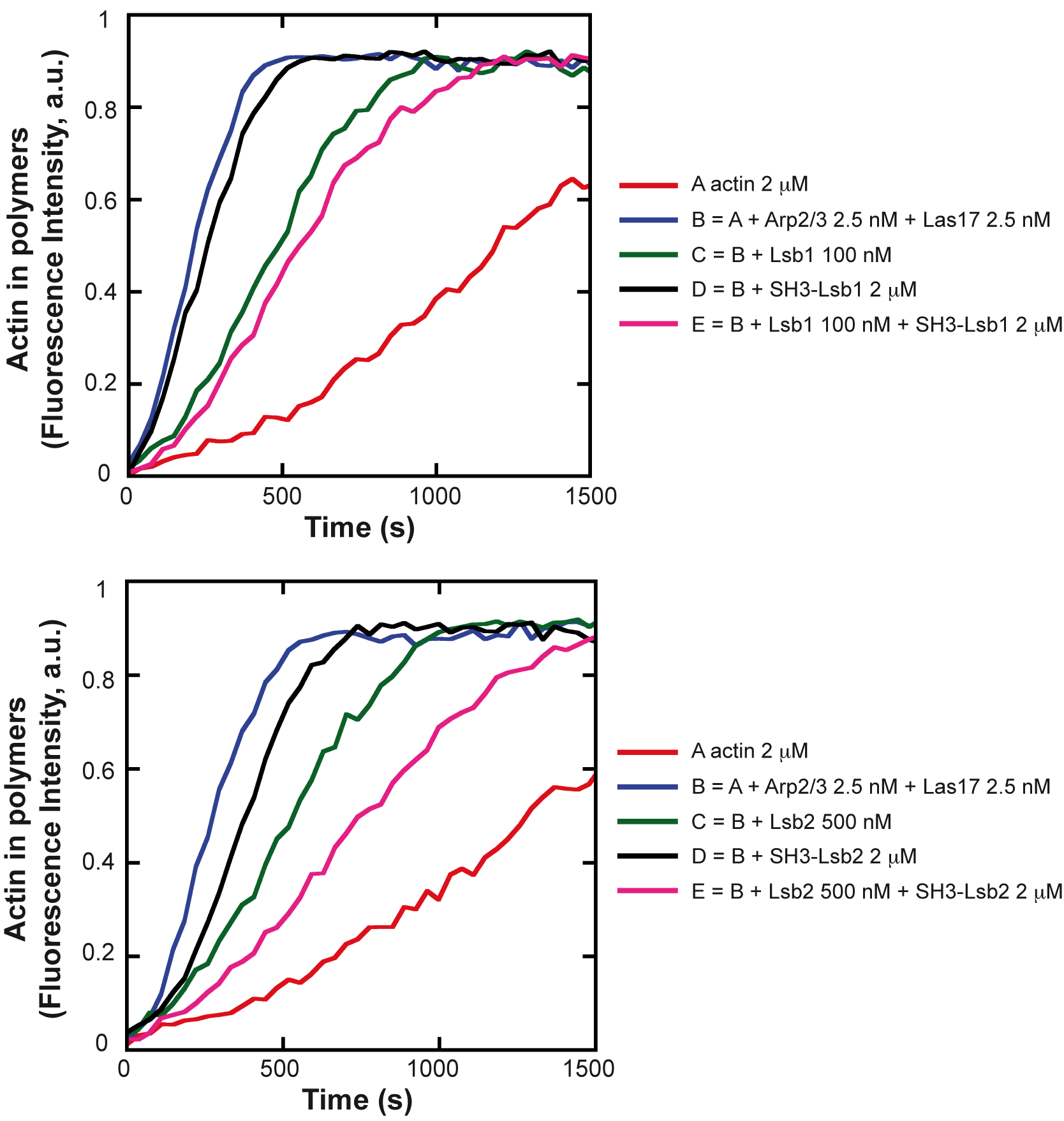

Supplement: Figure S2 — The SH3 domains of Lsb1 or Lsb2 proteins do not inhibit Las17 activity. A total of 2 µM of actin (3% pyrene labeled) was polymerized in the presence of indicated concentrations of recombinant purified Arp2/3 complex, Las17, Lsb1, Lsb2, SH3-Lsb1 and/or SH3-Lsb2 proteins. Actin polymers concentration expressed in arbitrary units (a.u.) was measured by the fluorescence of the pyrene-labeled actin. (TIF) [file pone.0061147.s002.tif]

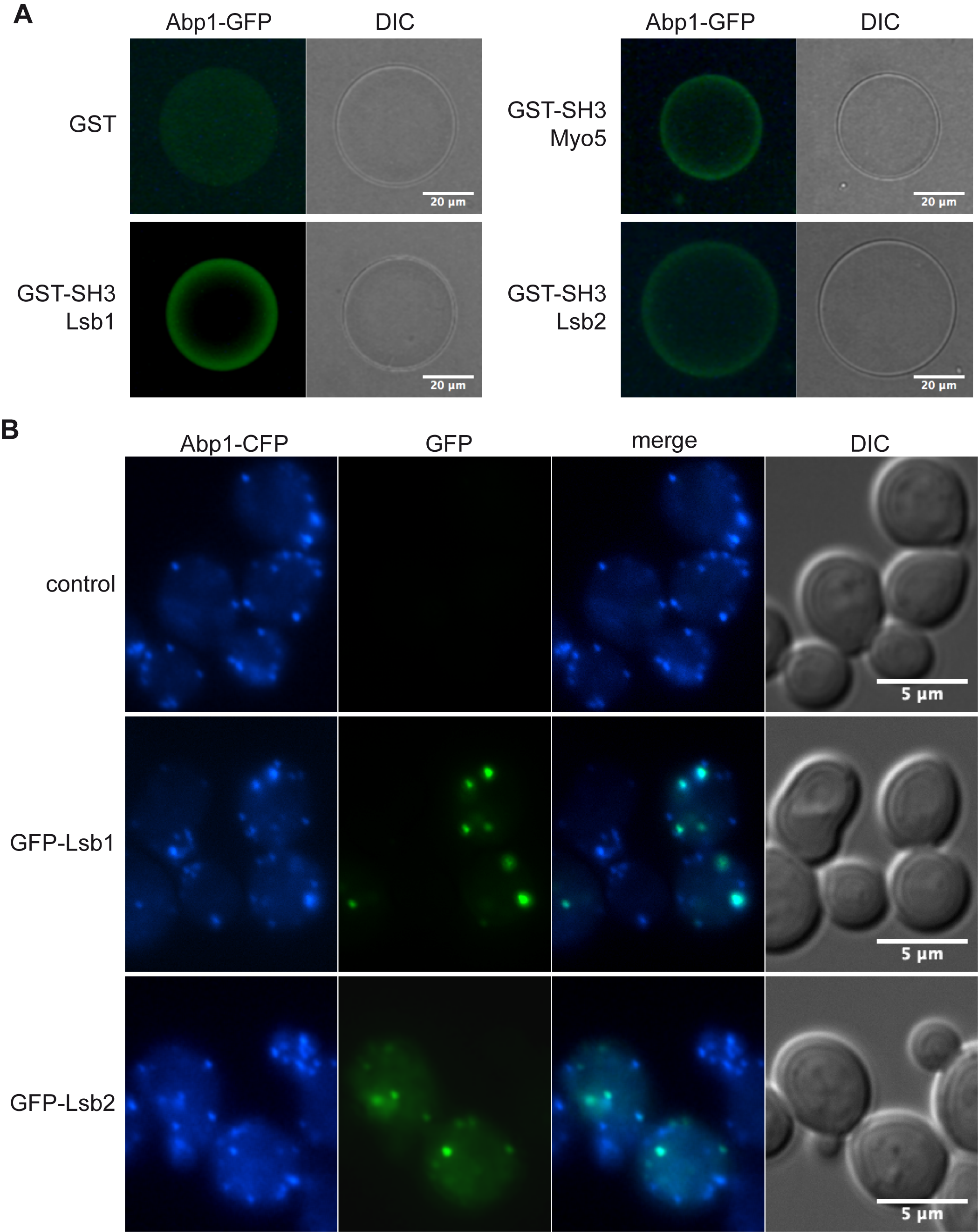

Supplement: Figure S3 — Lsb1 and Lsb2 colocalize with Abp1. A) GST, GST-SH3-Myo5, GST-SH3-Lsb1 and GST-SH3-Lsb2 proteins coated on glutathione Sepharose beads were incubated with a total protein extract from ABP1-GFP yeast cells. Beads were analyzed by fluorescence microscopy. GST was used as a negative control. B) The ABP1-CFP cells were transformed by pUG36-Lsb1 (GFP-Lsb1) or pUG36-Lsb2 (GFP-Lsb2) plasmids and the cells were observed by fluorescence microscopy. (TIF) [file pone.0061147.s003.tif]

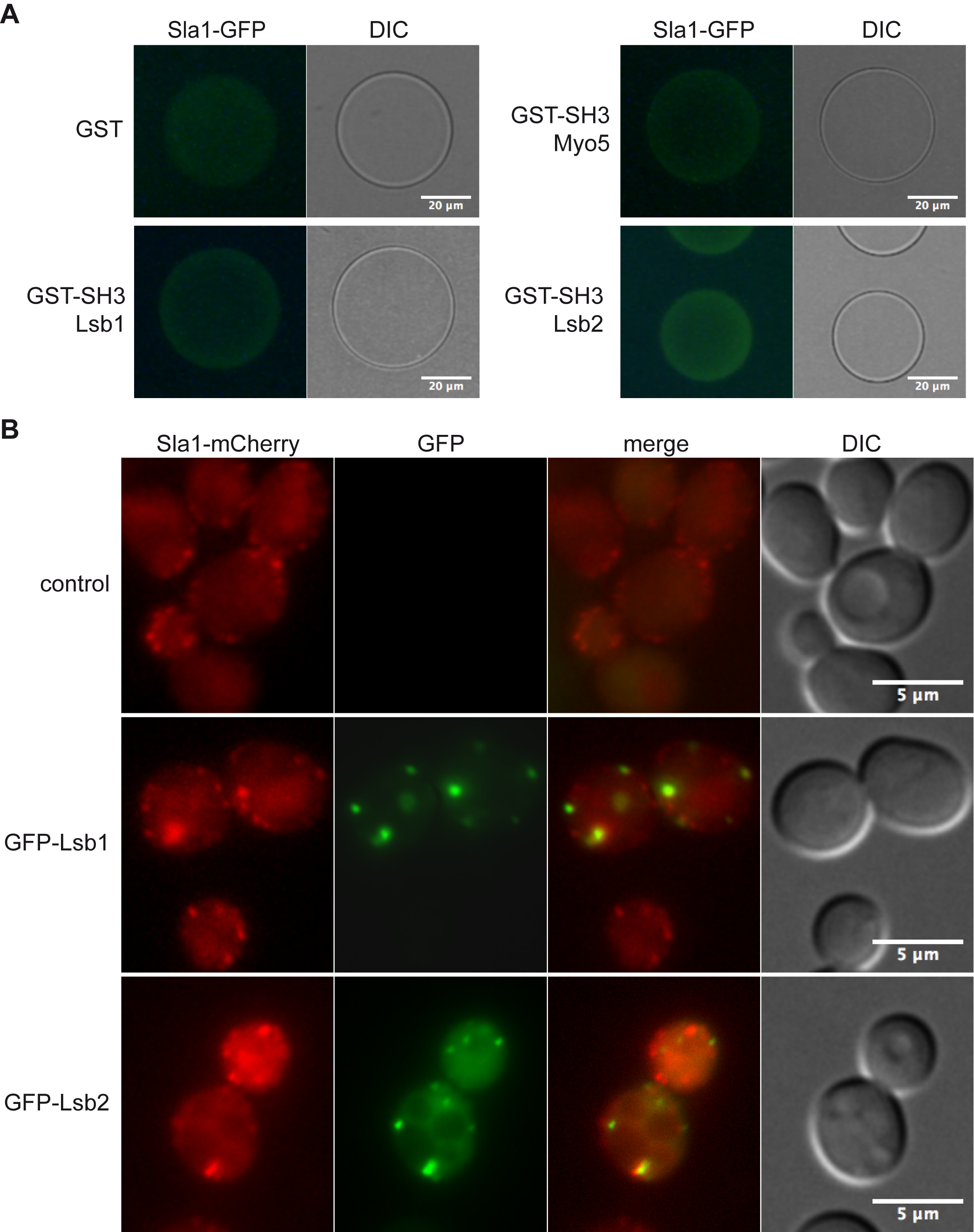

Supplement: Figure S4 — Lsb1 and Lsb2 colocalize with Sla1. A) GST, GST-SH3-Myo5, GST-SH3-Lsb1 and GST-SH3-Lsb2 proteins coated on glutathione Sepharose beads were incubated with a total protein extract from SLA1-GFP cells. Beads were analyzed using fluorescence microscopy. GST was used as a negative control. B) The SLA1-mCherry strain was transformed by pUG36-Lsb1 (GFP-Lsb1) or pUG36-Lsb2 (GFP-Lsb2) plasmids and the cells were observed by fluorescence microscopy. (TIF) [file pone.0061147.s004.tif]

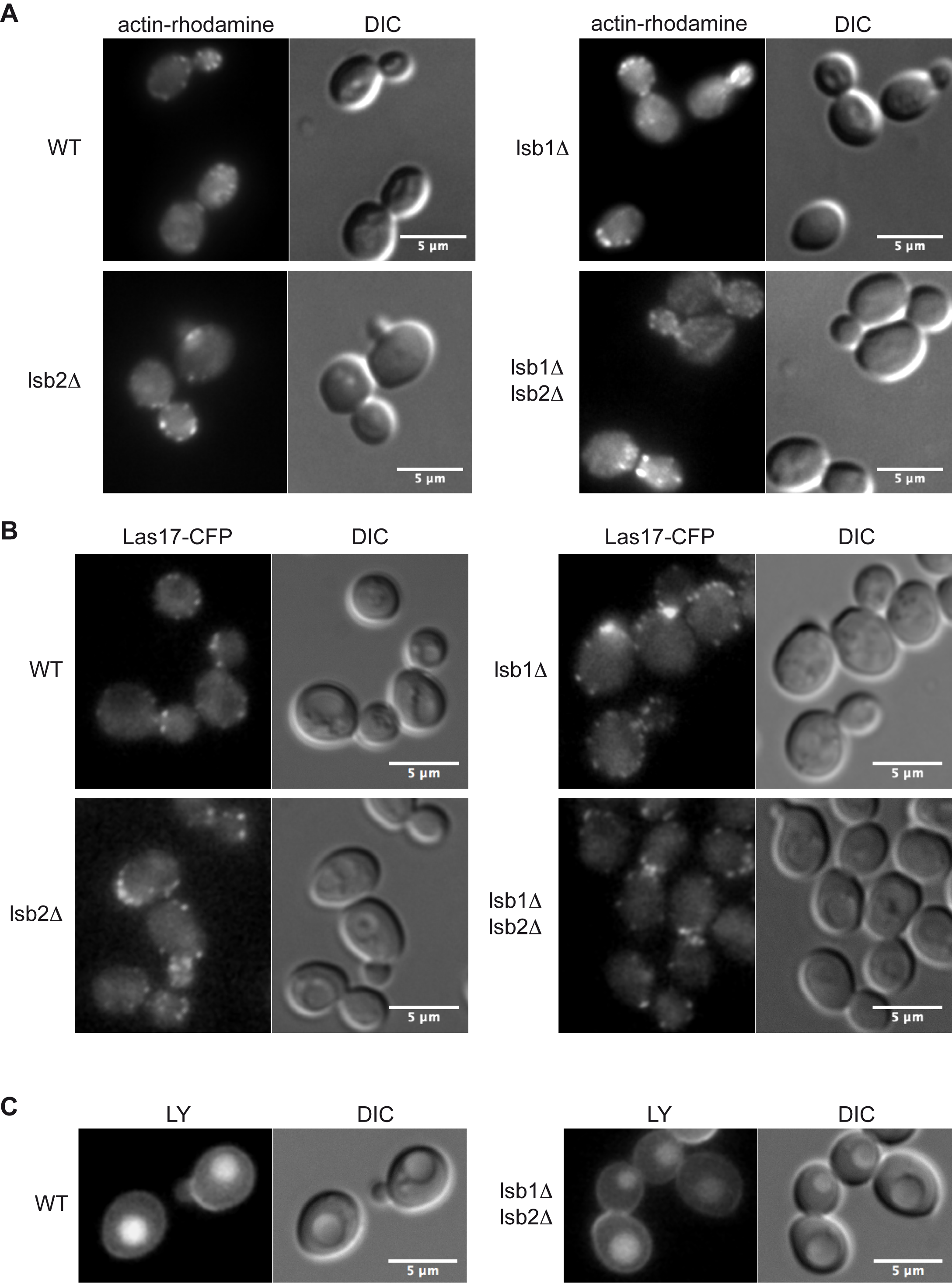

Supplement: Figure S5 — Las17 localized normal in a lsb1 Δ lsb2 Δ strain. A) The actin cytoskeleton was stained with phalloidin-rhodamine in wild type, lsb1Δ, lsb2Δ and lsb1Δ lsb2Δ cells prior observation by fluorescence microscopy. B) The exponentially growing LAS17-CFP, lsb1Δ LAS17-CFP, lsb2Δ LAS17-CFP and lsb1Δ lsb2Δ LAS17-CFP cells were observed by fluorescence microscopy. C) Wild type BY4742 and lsb1Δ lsb2Δ cells were analyzed by fluorescence microscopy after incubation for 60 min with the fluorescent dye Lucifer yellow (LY). LY is internalized via endocytosis and transported to the lumen of the vacuole. (TIF) [file pone.0061147.s005.tif]

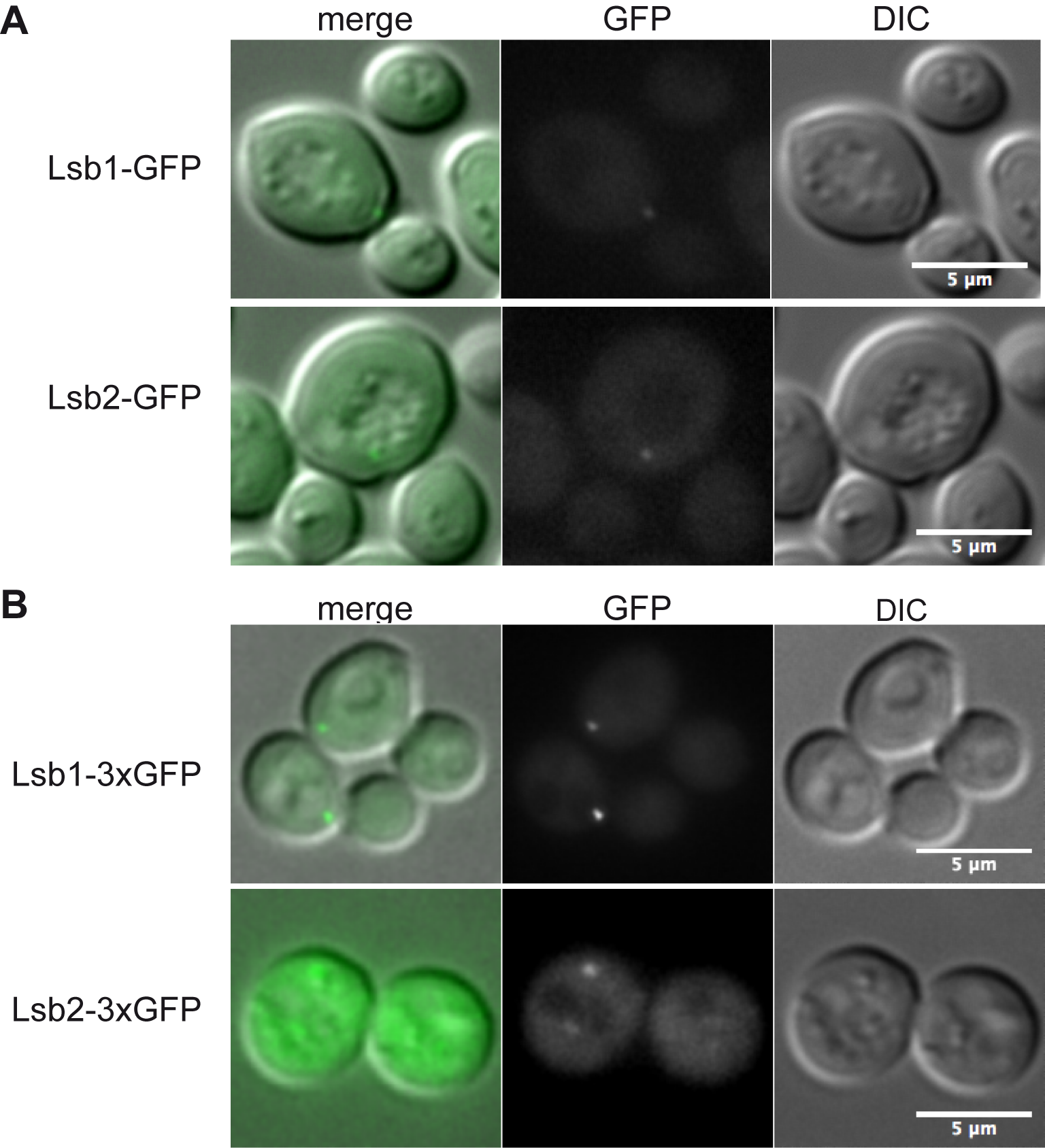

Supplement: Figure S6 — Lsb1 and Lsb2 expressed under their endogenous promoter localize to punctate structures. A) The BY4741 Lsb1-GFP and Lsb2-GFP cells were observed by fluorescence microscopy. B) Wild-type cells carrying a chromosomically integrated LSB1-3xGFP or LSB2-3xGFP fusion were analyzed for GFP fluorescence. (TIF) [file pone.0061147.s006.tif]

A

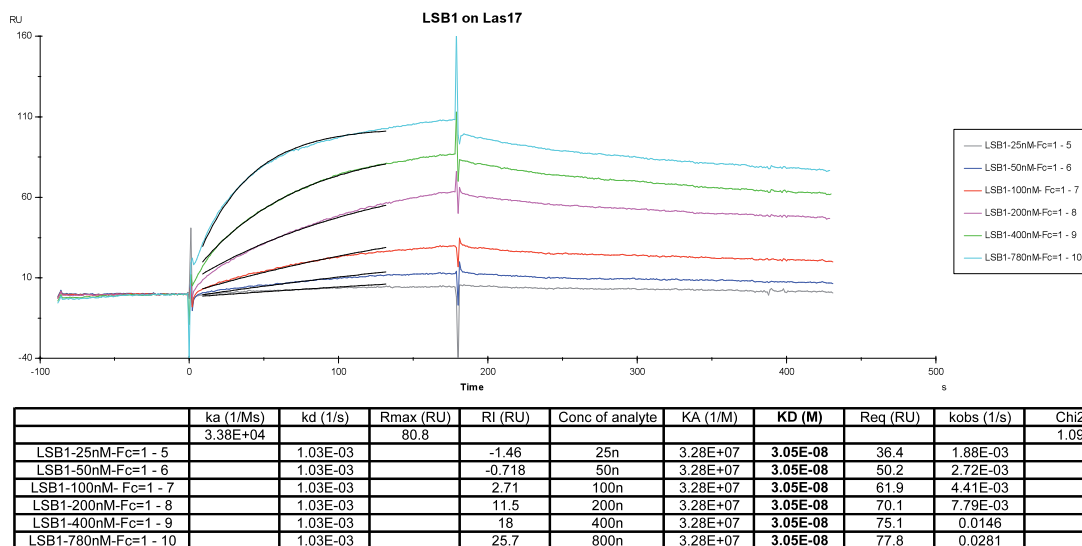

B

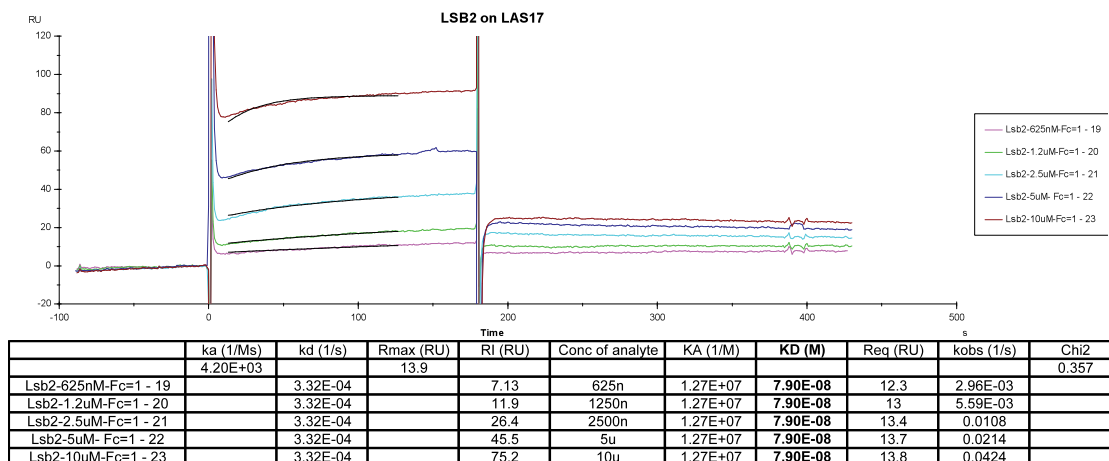

C

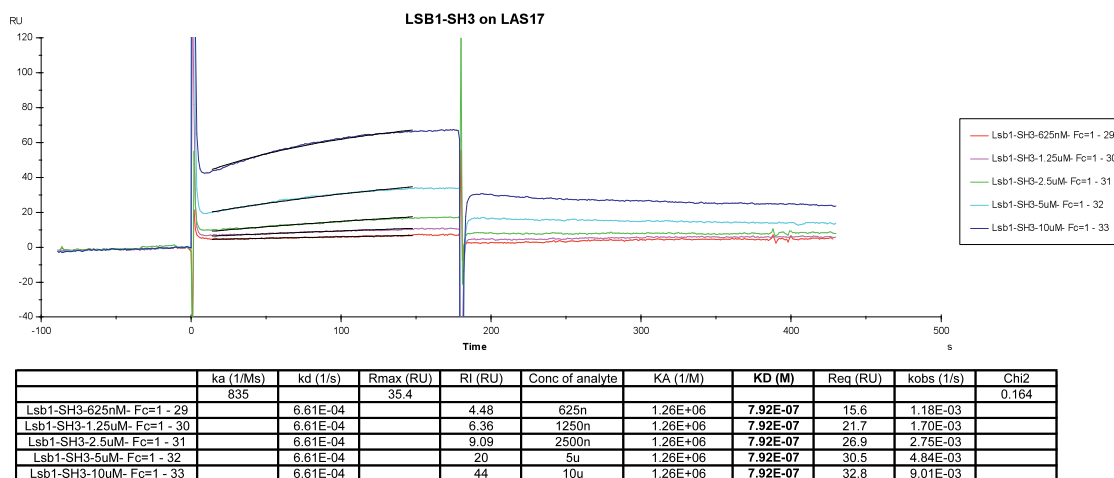

D

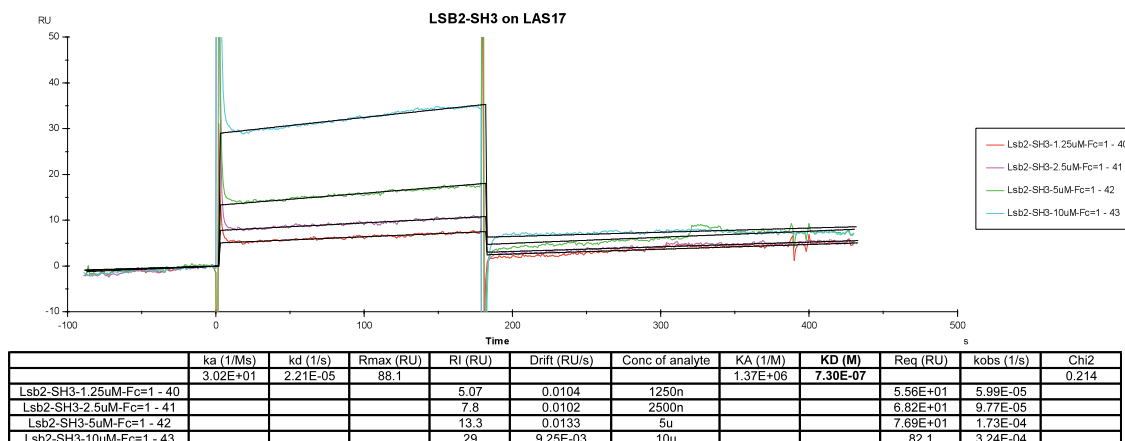

Supplement: Figure S7 — Biacore data of Lsb1 and Lsb2 with Las17. The raw data of measured KD constants obtained by SPR-based Biacore3000 measurements with Las17 and at different concentrations of (A) Lsb1, (B) Lsb2, (C) SH3-Lsb1 and (D) SH3-Lsb2 purified recombinant proteins. (PDF) [file pone.0061147.s007.pdf]
