# Supplementary material for: Lsb1 Is a Negative Regulator of Las17 Dependent Actin Polymerization Involved in Endocytosis
Source: PLoS One. 2013 Apr 8;8(4):e61147. doi: 10.1371/journal.pone.0061147 (PMC3620054; doi:10.1371/journal.pone.0061147)
Supplement: Table S1 — Strains and plasmids used in this study. (DOC) [file pone.0061147.s010.doc]

Table S1: Strains and Plasmids

| **Strain or Plasmid** | **Genotype or description** | **Reference** |
| --- | --- | --- |
| **Strains** |  |  |
| BY4742 | MAT**α** *his3Δ1 leu2Δ0 lys2Δ0 ura3Δ0* | Euroscarf |
| *LAS17-GFP* DDY2736 | MAT**α** *his3Δ200 leu2-3,112 lys2-801 ura3-52 LAS17-GFP:HIS3* | [37] |
| *LAS17-CFP* | MAT**α** *his3Δ1 leu2Δ0 lys2Δ0 met15Δ0 ura3Δ0 LAS17-CFP:KanMX* | This study |
| *lsb1*Δ *LAS17-CFP* | MAT**a** *his3Δ1 leu2Δ0 ura3Δ0 lsb1Δ::HIS3 LAS17-CFP:KanMX* | This study |
| *lsb2*Δ *LAS17-CFP* | MAT**a** *his3Δ1 leu2Δ0 lys2Δ0 ura3Δ0 lsb2Δ::KanMX LAS17-CFP:KanMX* | This study |
| *lsb1*Δ *lsb2*Δ *LAS17-CFP* | MAT**a** *his3Δ1 leu2Δ0 lys2Δ0 ura3Δ0 lsb1Δ::HIS3 lsb2Δ::KanMX LAS17-CFP:KanMX* | This study |
| *LSB1-GFP* | MAT**a** *his3Δ1 leu2Δ0 met15Δ0 ura3Δ0 LSB1-GFP:HIS3* | This study |
| *LSB2-GFP* | MAT**a** *his3Δ1 leu2Δ0 met15Δ0 ura3Δ0 LSB2-GFP:HIS3* | This study |
| *LSB1-3xGFP* | MAT**a** *his3Δ1 leu2Δ0 met15Δ0 ura3Δ0 LSB1-3xGFP:HIS3* | This study |
| *LSB2-3xGFP* | MAT**a** *his3Δ1 leu2Δ0 met15Δ0 ura3Δ0 LSB2-3xGFP:HIS3* | This study |
| *lsb1*Δ | MAT**a** *his3Δ1 leu2Δ0 ura3Δ0 met15Δ0 lsb1Δ::KanMX* | Euroscarf |
| *lsb2*Δ | MAT**a** *his3Δ1 leu2Δ0 lys2Δ0 ura3**Δ0 met15Δ0 lsb2Δ::KanMX* | Euroscarf |
| *lsb1*Δ *lsb2*Δ | MAT**a** *his3Δ1 leu2Δ0 met15Δ0 ura3Δ0 lsb1Δ::HIS3 lsb2Δ::KanMX* | This study |
| *las17*Δ | MAT**a** *his3-Δ200 leu2-Δ1 ura3-52 las17Δ::HIS3* | [22] |
| *SLA1-GFP* DDY2734 | MAT***a*** *his3Δ200 leu2-3,112 lys2-801 ura3-52 SLA1-GFP:HIS3* | [37] |
| *SLA1-mCh* DDY3743 | MAT***a*** *his3-Δ200 leu2-3,112 ura2-52 SLA1-mCherry:HIS3* | D.G. Drubin |
| *ABP1-GFP* DDY2733 | MAT***a*** *his3Δ200 leu2-3,112 lys2-801 ura3-52 ABP1-GFP:HIS3* | [37] |
| *ABP1-CFP* | MAT***a*** *leu2-3,112 lys2-801 ura3-52 ABP1-CFP:KanMX* | D.G. Drubin |
| **Plasmids** |  |  |
| pGST-Myo5-TH2,SH3 | C-terminal Myo5 fragment expressed as a GST fusion | [12] |
| pGST-Myo5-SH3 | The SH3 domain of Myo5 expressed as a GST fusion | This study |
| pGEX4-T1-Lsb1 | Lsb1 expressed as a GST fusion | This study |
| pGEX4-T1-Lsb2 | Lsb2 expressed as a GST fusion | This study |
| pGEX4-T1-SH3-Lsb1 | The SH3 domain of Lsb1 expressed as a GST fusion | This study |
| pGEX4-T1-SH3-Lsb2 | The SH3 domain of Lsb2 expressed as a GST fusion | This study |
| pRS416-Lsb1-HA | Lsb1-3xHA expressed under the native promoter | This study |
| pRS416-Lsb2-HA | Lsb2-3xHA expressed under the native promoter | This study |
| pUG36-Lsb1 | GFP-Lsb1 expressed under the *MET25* promoter | This study |
| pUG36-Lsb2 | GFP-Lsb2 expressed under the *MET25* promoter | This study |
| pUG-Lsb1-HA | Lsb1-3xHA expressed under the *MET25* promoter | This study |
| pUG-Lsb2-HA | Lsb2-3xHA expressed under the *MET25* promoter | This study |
| pFL91 | YCplac111-Can1promoter-CAN1-mRFP | [35] |
